# Association between a diagnostic journey in Angelman syndrome and caregivers’ quality of life

**DOI:** 10.1186/s11689-026-09693-1

**Published:** 2026-04-14

**Authors:** Jan Domaradzki, Dariusz Walkowiak

**Affiliations:** 1 Department of Social Sciences and Humanities, Poznan University of Medical Sciences, Rokietnicka 7, Poznań, 60-806 Poland; 2 Department of Organization and Management in Health Care, Poznan University of Medical Sciences, Poznań, Poland

**Keywords:** Angelman syndrome, Caregiver burden, Diagnostic odyssey, Family caregivers, Quality of life

## Abstract

**Background:**

Angelman syndrome (AS) is a rare neurogenetic disease that severely impacts a person’s health and daily living, requiring lifelong specialist care. This places substantial demands on caregivers, leading to a decline in physical and mental health, financial strain, and reduced quality of life. This study examines the association between a diagnostic journey in Angelman syndrome and parents’ quality of life.

**Methods:**

A self-administered, anonymous, computer-assisted online survey of 120 caregivers was conducted between March and August 2024. The Polish version of the WHO Quality of Life-BREF and the Consumer Financial Protection Bureau Financial Well-Being Scale were used. The collected information was imported into JASP 0.18.3.

**Results:**

54.2% of caregivers experienced difficulties in obtaining timely diagnoses, 51.6% reported receiving misdiagnoses, and 87.4% consulted several specialists before the correct diagnosis was confirmed. Many caregivers believed that a delayed diagnosis harmed their children’s health (31.7%) and resulted in unwarranted or inappropriate hospitalizations (18.3%), medical procedures (26.7%), and medications (16.7%). Caregivers struggled to access genetic counselling and psychological support. AS caregivers had lower quality of life scores compared to the national average across all domains, including social relationships (85.8%), physical health (80.8%), environmental (72.5%), and psychological (61.7%). Caregivers’ quality of life was associated with economic stability, gender, and the timing of diagnosis.

**Conclusion:**

There is a need for better access to genetic testing, a holistic healthcare approach, and comprehensive support services for caregivers, as well as reliable information, psychological support, financial assistance, and social services.

## Introduction

### Angelman syndrome

Angelman syndrome (AS, ICD-10: Q93.51, ICD-11: LD90.0, ORPHA: 72, OMIM: 105830) is a rare, genetically based disease that affects the nervous system, with an estimated prevalence between 1 in 10,000–24,000 live births [1, 2]. While it is caused by loss-of-function of the *UBE3A* gene on the maternal chromosome 15q11-q13, approximately 80% of cases of AS are caused by the deletion of the 15q11-q13 region, 10–11% of persons with AS have a mutation of the ubiquitin protein ligase E3A (*UBE3A*), and other causes include, paternal uniparental disomy (2–5%) and imprinting defect (2–5%) [3, 4]. This is important since it was reported that the molecular subtype of AS affects the degree and severity of symptoms in AS and the stress experienced by parents and informal caregivers [5, 6]. 

AS is characterized by a variety of neurological symptoms, which include moderate or profound intellectual disability and ataxia [7]. About 80–90% of persons with AS experience epilepsy, which usually manifests itself before the age of three and involves multiple seizure types [8]. Other associated comorbidities may include autistic spectrum disorder (ASD), cerebral palsy, scoliosis, gastrointestinal symptoms, and feeding problems [2, 9]. Lack of speech, sleep, and motor function impairments are also consistent features of AS. Behavioral symptoms include hyperactivity, disruptive behavior (pulling and grabbing, biting and scratching, hitting and kicking, yelling, hair pulling), frequent laughing, smiling, and excitability [4, 9–12]. Consequently, persons with AS require coordinated care and collaboration of various specialists [1] and experience a significant healthcare burden [13–16]. Especially since no approved disease-modifying treatment for AS is available, the treatment is purely symptomatic [17]. At the same time, life expectancy in individuals with AS may be similar to that in persons without the disease, but could be decreased by 10–15 years [18]. However, due to their excitement, some sudden deaths resulting from accidents, including drowning, were reported [19]. 

Because AS shares many symptoms with other disorders, many individuals are misdiagnosed [4, 11, 20, 21], and the AS diagnosis has to be confirmed by cytogenetic and molecular testing. The reason for this is that symptoms of AS are somehow similar to better-known conditions, including cerebral palsy, ASD, or Prader-Willi syndrome [9, 22–24]. 

### Caring for a person with Angelman syndrome

Because AS causes impairment of multiple systems, severely impacting an individual’s health, it puts substantial demands and challenges on family caregivers, leading to severe stress and a caregiving burden [6, 9, 12, 25]. AS influences caregivers’ daily routines, personal plans, family dynamics, and social relationships. Significantly, studies demonstrated that parents of children with AS experience higher levels of stress than both parents of children with other neurodevelopmental disorders and healthy children [5, 9, 26–32]. 

Thomson et al. reported that AS parents were stressed by a lack of quality information about their child’s disease, time constraints, and physical and emotional tiredness [30]. Van den Borne et al. showed that AS parents experienced a significant level of fear over negative consequences for themselves, including powerlessness and losing control. In contrast, parents of children with Prader-Willi syndrome (PWS) were more concerned about the negative health consequences for their children [31]. Another study demonstrated that while parents of children with several rare genetic syndromes experience anxiety, depression, and caregiving burden, parental stress and depression in both AS mothers and fathers were associated with children’s chronic challenging behavior [27, 28]. Similarly, Griffith et al. found that AS parents reported higher levels of clinical anxiety and depression than parents of children with Cornelia de Lange and Cri du Chat syndromes, autism, and without any syndrome [29]. Another study showed that AS parents experienced higher levels of stress and burden than parents of children with autism and without intellectual disability [27, 28]. Notably, AS caregivers’ quality of life (QoL) and perceived burden are affected by a prolonged diagnostic odyssey, which, on average, takes 1–4 years [9, 11]. 

To date, no study has been carried out on the QoL of Polish caregivers of persons with AS. This study is part of a broader project on the experiences of Polish caregivers of persons with rare diseases with the diagnostic odyssey. The aims were to (1) examine the experiences of AS caregivers with the diagnostic process and (2) determine the impact of the diagnostic journey on AS caregivers’ perceived QoL. We hypothesized that (1) AS patients experience a prolonged diagnostic process, and (2) the diagnostic odyssey in AS is associated with caregivers’ reduced QoL.

## Methods

### Study design

We designed a self-administered, anonymized, computer-assisted online survey to assess AS caregivers’ experiences with the diagnostic journey and its association with their perceived QoL. Since Poland lacks a registry of patients with rare diseases, and there is no official registry of AS patients in the country, to identify eligible participants, we contacted two advocacy organizations for AS patients and their families: FAST Poland - Foundation for Angelman Syndrome Therapeutics and the Association of Families with Angelman Syndrome (Stowarzyszenie Rodzin z Zespołem Angelmana), which helped in the recruitment process. While the survey was designed following the Declaration of Helsinki, it was approved by the Poznan University of Medical Sciences Bioethics Committee (KB – 228/24). Additionally, it received approval from both patient advocacy organizations involved in the study.

### Study population

To identify study participants, we contacted FAST Poland - Foundation for Angelman Syndrome Therapeutics and the Association of Families with Angelman Syndrome, which helped recruit caregivers via their website, Facebook pages, and newsletters. Participants were recruited if they were: (1) over 18 years old, (2) parents or family caregivers providing care to a person with a confirmed molecular diagnosis of AS, (3) were familiar with electronic devices to complete an online survey, (4) spoke Polish, and (5) provided written informed consent to participate.

### Questionnaire

The survey questionnaire was created with Microsoft Teams. It was divided into four sections. The first section included items regarding AS caregivers’ and patients’ socio-demographic data. The second section focused on caregivers’ experiences with the diagnostic process in their AS relatives. While these two parts of the questionnaire were designed as an ad hoc tool, they followed the guidelines of the European Statistical System. They were also evaluated by a panel of four experts: a medical sociologist, a public health specialist, a pediatrician specializing in rare diseases, and a mother of a child with a rare disease. They were also pre-tested on five parents of children with rare diseases.

The remaining two sections comprised two standardized research tools to measure caregivers’ QoL and financial well-being. The abbreviated Polish version of the WHO Quality of Life-BREF (WHOQOL-BREF) was employed to evaluate caregivers’ QoL [33, 34]. Although many years have passed since the Polish standardization, no newer version has emerged. However, due to the universality of this tool and its wide application, we decided to use the WHOQOL. This tool, widely utilized in epidemiological studies and clinical trials, is known for its reliability and contains 24 items divided into four domains: physical health, psychological health, social relationships, and environmental health. Respondents rated each item on a 5-point Likert scale. To assess financial well-being, we employed the Consumer Financial Protection Bureau Financial Well-Being Scale (CFPB-FWBS), a tool developed by the Consumer Financial Protection Bureau and known for its adaptability [35, 36]. 

### Procedure

The study was conducted over six months between March and August 2024, with two follow-up messages sent in May and June among caregivers of persons with AS associated with FAST Poland – Foundation for Angelman Syndrome Therapeutics and the Association of Families with Angelman Syndrome. It started by sending an invitation letter to participate in the survey. When permission was obtained, the research coordinator (JD) posted the online version of the questionnaire on Facebook, and all AS caregivers were invited to participate.

Due to the sensitive nature of the research topic, which might have created emotional distress in study participants, the online consent form, which stressed the anonymous, voluntary, and confidential character of the survey, was shown to all caregivers who volunteered to participate before they started the survey, asking them to select the “I agree” or “I do not agree” option. Filling out an online written consent form was required to access the questionnaire and be eligible to participate. All caregivers who volunteered provided written informed consent and did not receive financial compensation. Completing the survey took approximately 15–20 min.

### Statistical analysis

The collected information was carefully reviewed to ensure accuracy, consistency, and completeness before being processed and imported into JASP (Version 0.18.3) for comprehensive statistical analysis. The results are presented using descriptive statistical methods. Logistic regression models assessed differences in our respondents’ quality of life (QoL) across the four WHOQOL domains. Sociodemographic data served as explanatory variables, with the patient’s age with AS and the CFPB-FWBS score included as covariates. National average values [33, 34] were used as cut-off points within the WHOQOL domains, categorizing caregivers into two groups: those with scores below the average value for a given domain and those above the average.

The Cronbach’s alpha values calculated for the various domains were as follows: 0.773 for the WHOQOL Physical Health domain, 95% CI [0.703–0.830]; 0.826 for the WHOQOL Psychological domain, 95% CI [0.765–0.865]; 0.657 for the WHOQOL Social Relationships domain, 95% CI [0.536–0.752]; 0.785 for the WHOQOL Environment domain, 95% CI [0.720–0.837]; and 0.917 for CFPB-FWBS, 95% CI [0.892–0.937]. Although Cronbach’s alpha for the WHOQOL Social Relationships domain is somewhat lower, it is important to recognize that it comprises only three questions, one of which addresses sexual activity, which could confuse respondents in a highly conservative society. Nonetheless, our study group’s score for this domain was consistent with the scale’s validation process findings in Poland [33, 34].

## Results

One hundred twenty caregivers volunteered to take the survey. Participants enrolled in this study were predominantly mothers (71.7%) and were mainly aged 30–49 years (88.3%) (Table 1). A high proportion had university-level education (73.3%) and lived in cities with more than 10,000 inhabitants (36.7%). In addition, 39.2% were unemployed due to caregiving responsibilities. For comparison, according to Statistics Poland [37], the general unemployment rate in 2024 was 5.1%, and the employment rate among women was 51.5%. Additionally, 59% of the Polish population lives in urban areas. According to OECD data [38], approximately 39% of adults aged 25–64 in Poland have attained tertiary education. Compared with these reference values, the proportion of caregivers who were unemployed due to caregiving responsibilities (39.2%) and the proportion with university-level education (73.3%) in our sample appear higher than in the general population.

Among persons with AS, 49.2% were female, and 50.8% were male. Most children were between 6 and 10 years (45%) or 4 and 5 years (19.2%). Most frequently, the first symptoms appeared between the first age of the child’s life (83.3%), including 38.3% before 6 and 12 months, 25% between 2 and 5 months, and 20% within the first month of life. In most cases, general developmental, motor, neurological, and cognitive symptoms led to the diagnostic process (67.5%, 60%, 56.7% and 36.7%, respectively).

**Table 1:** Socio-demographic characteristics of caregivers and persons with AS

AS caregivers		Persons with AS	
Characteristic	*N* (%)	Characteristic	*N* (%)
*Sex*		*Sex*	
woman	86 (71.7)	female	59 (49.2)
man	34 (28.3)	male	61 (50.8)
*Relationship with an AS patient*		*Age*	
mother	86 (71.7)	0–1	4 (3.3)
father	33 (27.5)	2–3	6 (5)
legal guardian	1 (0.8)	4–5	23 (19.2)
*Age*		6–10	54 (45)
20–29	5 (4.2)	11–15	15 (12.5)
30–39	64 (53.3)	16–18	7 (5.8)
40–49	42 (35)	19–20	4 (3.3)
50–59	8 (6.7)	above 20	7 (5.8)
60–69	1 (0.8)		
*Domicile*		*Age at which the first symptoms appeared*	
up to 10,000 inhabitants	38 (31.7)	the prenatal period	3 (2.5)
10–50,000 inhabitants	20 (16.7)	the neonatal period (first month of life)	24 (20)
51–100,000 inhabitants	18 (15)	2–5 months of age	30 (25)
101–500,000 inhabitants	27 (22.5)	6–12 months of age	46 (38.3)
above 500,000 inhabitants	17 (14.2)	1–3 years of age	16 (13.3)
		4-5years of age	1 (0.8)
*Education*		Type of symptoms that initiated the diagnostic process*	
primary school	1 (0.8)	general development	81 (67.5)
vocational school	11 (9.2)	motor	72 (60)
high school	20 (16.7)	neurological	68 (56.7)
university	88(73.3)	cognitive	44 (36.7)
		gastrointestinal (digestive)	32 (26.7)
*Professional activity*		cardiovascular	3 (2.5)
unemployed due to caregiving	47 (39.2)	psychiatric	3 (2.5)
employed part-time	17 (14.2)	immunological	1 (0.8)
employed full-time	56 (46.7)	nephrological	1 (0.8)
		other	3 (2.5)

^*^It was possible to give more than one answer

The characteristics of the diagnostic journey, as reported by caregivers, are presented in Table 2. While most caregivers indicated that their children were diagnosed between one and three years of age (62.5%), 18.3% reported that the diagnosis was made after the age of four. Diagnostic delays were common: 35% stated that the process lasted between one and two years, and 19.2% indicated that it exceeded three years. Although 48.3% reported no prior misdiagnosis, 51.7% stated that their child had received at least one incorrect diagnosis. The majority of caregivers (65.8%) reported consulting two to four specialists before the correct diagnosis was confirmed, and 21.6% reported visiting five or more specialists. While 89.2% indicated having contact with a genetic clinic, only 33.3% reported that Whole Exome Sequencing was performed. Psychological counselling during the diagnostic process was reported by only 6.7% of caregivers.

**Table 2:** Diagnostic journey in AS

Characteristics	*N* (%)
*Age of diagnosis*	
till the 5th month of life	5 (4.2)
till the 12th month of life	18 (15)
1–3	75 (62.5)
4–5	12 (10)
6–10	4 (3.3)
11–15	5 (4.2)
16–18	0 (0)
over 18	1 (0.8)
*Time spent before the correct diagnosis was made*	
1–3 months	6 (5)
4–6 months	15 (12.5)
7–12 months	34 (28.3)
1–2 years	42 (35)
3–5 years	16 (13.3)
6–7 years	2 (1.7)
8–10 years	2 (1.7)
11–15 years	3 (2.5)
*Number of misdiagnoses*	
0	58 (48.3)
1	28 (23.3)
2–3	31 (25.8)
4–5	2 (1.7)
6–7	1 (0.8)
*The number of specialists consulted*	
1	15 (12.5)
2–4	79 (65.8)
5–7	19 (15.8)
8–10	4 (3.3)
11–15	3 (2.5)
*Had contact with a genetic clinic during the diagnosis*	
yes	107 (89.2)
no	13 (10.8)
*A Whole Exome Sequencing test was performed*	
yes	40 (33.3)
no	80 (66.7)
*Source of funding for diagnostic tests*	
the National Health Fund	72 (60)
participation in experimental research, university/hospital research grant	6 (5)
private financial resources	38 (31.7)
private and external help	4 (3.3)
*Received psychological counselling*	
yes	8 (6.7)
no	112 (93.3)
*Seeking help abroad at any stage of diagnostics*	
yes	22 (18.3)
no	98 (81.7)

While 55% of caregivers declared that misdiagnosis or late diagnosis did not harm their child’s health, a significant portion believed it had a damaging effect (31.7%) (Table 3). Similarly, although 83.3% of caregivers reported that misdiagnosis or late diagnosis did not result in unnecessary or inappropriate medication uptake, 16.7% suggested it did. While most caregivers (81.7%) said their child had not been hospitalized for unwarranted or inappropriate reasons because of a misdiagnosed or delayed diagnosis, 18.3% reported at least one such hospital stay. Moreover, 23.3% of caregivers suggested that the diagnostic odyssey resulted in unnecessary tests, treatments, or surgeries in their children with AS. Finally, even though 73.3% of caregivers reported that their child had no excessive or inappropriate medical procedures, including diagnostic tests, therapies, or operations that resulted from misdiagnosis or late diagnosis, 26.7% reported at least one such procedure.

**Table 3:** AS caregivers’ opinions on the consequences of the diagnostic journey on a child’s health

	*N* (%)
Misdiagnosis/late diagnosis has hurt a child’s health	
definitely yes	20 (16.7)
rather yes	18 (15)
I do not know	16 (13.3)
rather not	60 (50)
definitely not	6 (5)
*Number of unnecessary/inappropriate medications taken by a child as a result of misdiagnosis/late diagnosis*	
0	100 (83.3)
1–3	17 (14.2)
4–6	2 (1.7)
7–10	1 (0.8)
*Number of unnecessary/inappropriate hospitalizations resulting from misdiagnosis/late diagnosis*	
0	98 (81.7)
1–3	18 (15)
4–6	3 (2.5)
7–10	1 (0.8)
*Did your sick relative undergo unnecessary tests*,* treatments*,* or surgeries as a result of previous inappropriate diagnoses?*	
yes	28 (23.3)
no	82 (68.3)
I do not know	10 (8.3)
*Number of unnecessary/inappropriate medical procedures (i.e.*,* diagnostic tests*,* therapies*,* operations) resulting from misdiagnosis/late diagnosis*	
0	88 (73.3)
1–3	22 (18.3)
4–6	8 (6.7)
7–10	2 (1.7)

The respondents scored below the country’s mean in all WHOQOL domains (Table 4). In the Physical health domain, 80.8% of respondents scored below the national mean, showing a significant deviation from the population distribution (*p* < 0.001). In the Psychological health domain, 61.7% of caregivers scored below the mean (*p* = 0.01). In the Social relationships domain, 85.8% of respondents scored below the mean (*p* < 0.001). Lastly, 72.5% of caregivers scored below the national mean in the Environment domain (*p* < 0.001).

**Table 4:** Distribution of respondents’ scores relative to the mean country score in the WHOQOL domains

WHOQOL domain	Number of Respondents with Score Below Mean Country Score *N* (%)	Number of Respondents with Score Above Mean Country Score *N* (%)	*p*
Physical health	97 (80.8)	23 (19.2)	< 0.001
Psychological health	74 (61.7)	46 (38.3)	0.01
Social relationships	103 (85.8)	17 (14.2)	< 0.001
Environment	87 (72.5)	33 (27.5)	< 0.001

Table 5 presents Spearman’s correlations between the WHOQOL-BREF domains, indicating significant positive correlations across all pairings. The correlation between the Physical health and Psychological health domains was the strongest, with a Spearman’s rho of 0.694 (95% CI: 0.591–0.783, *p* < 0.001). The Physical health and Environment domains also strongly correlated with a Spearman’s rho of 0.624 (95% CI: 0.489–0.731, *p* < 0.001). The correlations between the Physical health and Social relationships domains, as well as between the Psychological healthand Social relationships domains, were moderate, with Spearman’s rho values of 0.457 (95% CI: 0.294–0.595, *p* < 0.001) and 0.593 (95% CI: 0.464–0.712, *p* < 0.001), respectively. The Psychological health and Environment domains had a Spearman’s rho of 0.648 (95% CI: 0.524–0.760, *p* < 0.001), and the Social relationships and Environment domains were also significantly correlated, with a Spearman’s rho of 0.571 (95% CI: 0.420–0.692, *p* < 0.001). All confidence intervals were calculated based on 1,000 bootstrap replicates, confirming the robustness of these correlations.

**Table 5:** Spearman’s correlations between WHOQOL-BREF domains

	Spearman’s rho	Lower 95% CI	Upper95% CI	*p*
Physical health -Psychological health	0.694	0.591	0.783	< 0.001
Physical health -Social relationships	0.457	0.294	0.595	< 0.001
Physical health -Environment	0.624	0.489	0.731	< 0.001
Psychological health-Social relationships	0.593	0.464	0.712	< 0.001
Psychological health-Environment	0.648	0.524	0.760	< 0.001
Social relationships- Environment	0.571	0.420	0.692	< 0.001

Confidence intervals based on 1000 bootstrap replicates

The regression analysis results for the WHOQOL domains across different models indicate significant findings (Table 6). The intercepts in all models are highly significant, with particularly low odds ratios in Models 1.1, 1.2, and 1.3. The CFPB-FWBS variable consistently shows a significant positive association with WHOQOL domains, with odds ratios between 1.050 and 1.178. Gender differences reveal that males have significantly higher odds ratios in Models 1.2 and 1.3, at 2.954 and 3.326, respectively. Compared to late diagnosis, early diagnosis exhibits a significant positive association in Model 1.3, with an odds ratio of 3.076. However, employment status (non-working vs. working) does not show a significant association in the model where it is included. The McFadden R-squared values vary, with the highest at 0.299, while the Nagelkerke R-squared values range from 0.089 to 0.429.

**Table 6:** Regression analysis results for WHOQOL domains

Regression parameters	WHOQOL Physical health			WHOQOLPsychological health	WHOQOL Social relationships	WHOQOL Environment	
	Model 1.1	Model 1.2	Model 1.3	Model 2.1	Model 3.1	Model 4.1	Model 4.2
	OR (SE)	OR (SE)	OR (SE)	OR (SE)	OR (SE)	OR (SE)	OR (SE)
Intercept	0.003^***^ (1.483)	0.002^***^ (1.528)	4.499 × 10^−4 ***^ (1.763)	0.044^**^ (0.998)	0.165^***^ (0.262)	1.034 × 10^−4 ***^ (1.756)	5.804 × 10^−5 ***^ (1.866)
CFPB-FWBS	1.083^**^ (0.025)	1.082^**^ (0.025)	1.096 ^***^ (0.027)	1.050^**^ (0.018)		1.154^***^ (0.029)	1.178^***^ (0.033)
Male vs. female		2.954^*^ (0.513)	3.326 ^*^ (0.531)				
Early diagnosis vs. late diagnosis			3.076 ^*^ (0.557)				
Non-working vs. working							0.350 (0.574)
Observations	120	120	120	120	120	120	120
R2 McFadden	0.107	0.145	0.182	0.051	0.000	0.274	0.299
R2 Nagelkerke	0.159	0.211	0.262	0.089		0.398	0.429
AIC	108.742	106.316	103.898	155.607	99.915	106.290	104.939
p-value for Model	< 0.001	0.035	0.036	0.004		< 0.001	0.060

^*^p-value < 0.05, ^**^p-value < 0.01, ^***^p-value < 0.001

## Discussion

This article reports several important findings. Firstly, it indicates that although the genetic foundation of AS has been identified and extensively documented, and genetic testing is available [1, 3, 4], children with AS encounter various difficulties during the diagnostic process. While a substantial portion of parents reported difficulties in receiving a correct and timely diagnosis for their children with AS, 51.6% reported receiving a misdiagnosis. However, Suleja et al. also demonstrated that while the mean age when children with AS received a diagnosis was 2.4 years, the diagnostic process took, on average, 14 months. It was also demonstrated that while the first concerning symptoms are typically seen by caregivers around five months, it takes approximately 12 months to receive a referral for genetic testing [11]. 

This is hardly surprising given that previous studies reported that Polish healthcare professionals, including doctors and nurses, have insufficient knowledge of rare diseases and are unprepared to care for such patients [39], who often experience the diagnostic odyssey. For example, while in 54% of Polish children with Dravet syndrome, the diagnostic process took up to 3 years, and in 5.4%, more than 10 years [40], the average diagnostic period for people with Huntington’s disease was 10.5 years (range 1–30) [41]. Similarly, a recent study showed that the diagnostic journey in children with rare and ultra-rare diseases lasted, on average, 2.5 years, and in many cases, it took 10 or even 18 years [42]. 

Secondly, this study confirms that while caregivers have difficulties in getting a diagnosis for their children with AS, they often need to consult several specialists before the correct diagnosis is confirmed. However, previous studies also showed that many patients with AS receive the incorrect diagnosis [4, 9, 20, 22, 24]. For example, Roche et al. reported that 48% of parents declared that before being diagnosed with AS, their children had been misdiagnosed with autism, developmental delay, epilepsy, cerebral palsy, brain injuries, or ADHD, hypotonia, PWS, Rett Syndrome, or other rare diseases. Misdiagnoses or differential diagnoses have been particularly common among children who had not been diagnosed with AS by 3 years of age [21]. Similarly, Suleja et al. demonstrated that, before being diagnosed with AS, 37.9% of Polish children were misdiagnosed with other conditions such as cerebral palsy, West syndrome, Cri-du Chat syndrome, spinal muscular atrophy, Dravet syndrome, or PWS. Significantly, while in children without a misdiagnosis, the average time to diagnosis was 10 months, in those with a previous misdiagnosis, this time was twice as long and lasted, on average, 22 months, leading to delayed initiation of the proper course of treatment [11]. 

Thirdly, this study reveals that nearly one-third of caregivers felt that a delayed diagnosis harmed their children’s health, resulting in many unwarranted or inappropriate hospitalizations, medical procedures, and medications. Furthermore, most caregivers expressed difficulties in obtaining genetic counselling and psychological support. Thus, it confirms the observation made by Suleja et al., who demonstrated that while, on average, a clinical geneticist appointment took three months to schedule, 64.3% of parents of children with AS reported that the biggest challenge was finding the right specialist to identify abnormalities and recommend genetic testing [11]. 

Fourthly, this study shows that while the quality of life for AS caregivers is generally low, it is also significantly lower than the average country score [33, 34]. Moreover, while most caregivers scored below the mean country score across all four QoL domains, their experiences were mainly associated with their economic stability, gender, and the timing of their diagnosis [26]. This is consistent with Hagenaar et al., who found that the impact of the child’s syndrome on the parent, measured with the Infant and Toddler Quality of Life Questionnaire (ITQOL), was in the “exceptionally low” range relative to normative data, indicating a very high level of parental burden. Emotional and behavioural problems were the strongest predictors of child health-related quality of life, which was exceptionally low. In contrast, parenting stress was above average [5]. Similarly, a recent study conducted in Portugal confirmed the presence of high levels of burden among AS parents. It was also demonstrated that while caregivers experienced many negative emotions, including sadness, shock, fear, and uncertainty, they were mainly burdened by patients’ lack of autonomy, the diversity of caregiving tasks, and a lack of time for themselves. At the same time, parents who were more satisfied with their caregiving role reported lower burden levels [43]. 

Other studies also found that AS parents’ quality of life was associated with their night-time sleep, which was, in turn, disturbed by their children/adolescents’ sleep problems, i.e., high nocturnal waking and arousal [5, 44], behavioural and emotional issues [7, 8], and motor and functional dependency [6, 28]. Moreover, previous findings demonstrated that both parents of children with AS reported higher levels of stress than parents of children with other neurological disorders [31, 45]. For example, Wulffaert et al. showed that mothers of children with AS reported higher levels of parenting stress than mothers of children with PWS (58% vs. 26%) [32]. Similarly, Adams et al. found that from 13 different genetic syndromes, AS is amongst those whose mothers are the most stressed. Moreover, they also reported significantly lower positive gains from caregiving [28]. 

Finally, this study showed a negative correlation between AS caregivers’ perception of the diagnostic odyssey and quality of life. This finding aligns with previous studies that have reported that AS caregivers feel burdened by the prolonged diagnostic journey, which usually takes between 1 and 4 years [9]. Similarly, mothers of adult offspring with several rare diseases, including AS, reported struggling to receive appropriate social care and medical services [13]. At the same time, while parents and caregivers are frustrated and dissatisfied with the attitudes of healthcare staff, including how they pass parents information about their child’s diagnosis, they also stress that accurate and timely information during the diagnostic period helps reduce stress [30, 31]. Our finding also aligns with the observation made by van den Borne et al. [31], who found that parents of children with AS reported having significant needs regarding where and how to get information about raising their child. Also, Thomson et al. demonstrated that a lack of adequate information at the time of initial diagnosis was one of the most stressful experiences for AS parents, leading to many negative emotions, including disappointment, hopelessness, and anger [30]. 

In addition to the observed associations between diagnostic experience and QoL, it is important to consider the relationships among the QoL domains themselves. The pattern of inter-domain correlations observed in our study is consistent with prior validation research on the WHOQOL-BREF conducted in both the general population and clinical samples. In the original international field trial led by the World Health Organization WHOQOL Group, correlations between domains ranged from 0.46 to 0.67, with the strongest association typically observed between the Physical health and Psychological health domains [46]. Epidemiologically ascertained samples have reported similar or even higher coefficients, suggesting that moderate to strong inter-domain associations represent a stable and replicable characteristic of the instrument rather than a population-specific artifact [47–50]. In the present study, Spearman’s rho coefficients ranged from 0.457 to 0.694, closely paralleling the magnitude and configuration described in previous research. Notably, the strongest relationship was again observed between the Physical health and Psychological health domains, which is theoretically plausible given the well-established bidirectional relationship between somatic functioning and emotional well-being.

From a methodological perspective, the observed coefficients do not reach levels typically considered indicative of redundancy or lack of discriminant validity (e.g., > 0.85–0.90) [51–53]. Rather, the pattern supports the conceptual model underlying the WHOQOL-BREF, in which domains represent distinguishable yet inherently interrelated facets of overall quality of life. Contemporary theories of quality of life conceptualize it as a multidimensional construct characterized by partial domain overlap due to shared variance attributable to global well-being. Within this framework, moderate inter-domain correlations are expected and reflect construct coherence rather than structural weakness [54, 55]. Nevertheless, while our findings are consistent with prior validation studies, we acknowledge that high inter-domain correlations always warrant careful interpretation. Future studies employing confirmatory factor analysis or alternative approaches to assessing discriminant validity (e.g., HTMT ratios) could further elucidate the degree of structural distinctiveness among domains in AS populations.

The key strength of this study is that, as far as we know, this is the first study on the experiences of Polish AS caregivers. Additionally, it included responses from 120 caregivers, which constitutes one of the largest national survey-based samples of caregivers of individuals with AS reported to date. Previous studies in this field have typically included smaller samples, often ranging from approximately 15 to 60 participants in single-country studies, while larger registry-based or multinational studies have included around 120–180 respondents. Importantly, according to the Global Angelman Syndrome Registry, 145 individuals from Poland are currently registered [56]; therefore, our sample represents a substantial proportion of the potentially reachable national population. Furthermore, it highlights the main challenges AS caregivers face during the diagnostic journey with their relatives and sheds light on the impact of the diagnostic odyssey on caregivers’ perceived quality of life. Thus, this study fills a gap in research on the situation of Polish AS caregivers and may stimulate further investigation into factors affecting caregivers of individuals with AS in Poland. Finally, since many respondents indicated that it was the first time they had an opportunity to speak about their experiences as caregivers of a person with AS, the study may also have had therapeutic value.

At the same time, this study has some limitations that should be acknowledged. Most importantly, although 120 caregivers completed the survey, the actual number of AS caregivers in Poland is likely higher. However, since there is no national registry of rare diseases, the precise number of individuals with AS and their families remains unknown. Additionally, as the data were collected via websites and Facebook pages of patient organizations, there is a risk of recruitment bias, as not all caregivers may be affiliated with such groups. Similarly, although 34 fathers responded, there is also a gender bias, as mothers predominated. Moreover, very few caregivers of adult individuals with AS responded. The self-reported and subjective character of the survey also limits this study, as all information regarding the diagnostic process and clinical history was based on caregivers’ reports and was not verified against medical documentation, which may introduce recall bias.

Although WHOQOL-BREF is a well-validated instrument, the present study did not include a comparison group nor conduct a confirmatory factor analysis within this specific caregiver population; therefore, the interpretation of inter-domain correlations should be approached with caution. Additionally, due to the cross-sectional design, causal inferences cannot be drawn. We did not collect detailed clinical data (e.g., molecular subtype, comorbidities, or symptom severity), nor did we assess AS genotype, although previous research suggests that genotype may influence symptom severity and caregiver stress. As medical records were not reviewed, it cannot be fully excluded that some respondents may have reported on individuals without a confirmed AS diagnosis. A part of the questionnaire used in this survey was not validated. Finally, since this study was designed as a quantitative survey, in-depth qualitative research could further enhance understanding of caregivers’ perspectives.

## Conclusions

These results can direct the development of support programs for AS families and guide the intervention approach. We recommend that the following policies be implemented:

1. Genetic testing should be widely available and simple to obtain.
2. Given the significant diagnostic delay observed in AS and the potential benefits of early multidisciplinary intervention, inclusion of AS in future newborn screening programs may be considered, particularly in the context of emerging targeted therapies.
3. Since many patients with AS suffer from misdiagnosis and contact numerous specialists, doctors should be trained about the nature of AS.
4. Throughout the entire diagnosis process and the lifecycle, AS caregivers should be provided with timely and reliable information regarding their child’s disease, prognosis, available treatments, and resources for support.
5. Caregivers of children with AS should have access to psychological assistance and financial support.
6. Caregivers of children with AS should have continuous access to services, including respite care and accommodation support.

## Data Availability

The data underlying this study’s findings cannot be shared publicly due to the sensitivity and privacy of the study participants. The data will be shared at a reasonable request by the corresponding author.

## Abbreviations

AS: Angelman syndrome
ASD: Autistic spectrum disorder
PWS: Prader-Willi syndrome
QoL: Quality of life
